# Traditional Norwegian Kveik Are a Genetically Distinct Group of Domesticated *Saccharomyces cerevisiae* Brewing Yeasts

**DOI:** 10.3389/fmicb.2018.02137

**Published:** 2018-09-12

**Authors:** Richard Preiss, Caroline Tyrawa, Kristoffer Krogerus, Lars Marius Garshol, George van der Merwe

**Affiliations:** ^1^Department of Molecular and Cellular Biology, University of Guelph, Guelph, ON, Canada; ^2^Escarpment Laboratories, Guelph, ON, Canada; ^3^VTT Technical Research Centre of Finland, Espoo, Finland; ^4^Department of Biotechnology and Chemical Technology, School of Chemical Technology, Aalto University, Espoo, Finland; ^5^Independent Researcher, Rælingen, Norway

**Keywords:** yeast, domestication, brewing, *Saccharomyces*, fermentation, kveik, ale

## Abstract

The widespread production of fermented food and beverages has resulted in the domestication of *Saccharomyces cerevisiae* yeasts specifically adapted to beer production. While there is evidence beer yeast domestication was accelerated by industrialization of beer, there also exists a farmhouse brewing culture in western Norway which has passed down yeasts referred to as kveik for generations. This practice has resulted in ale yeasts which are typically highly flocculant, phenolic off flavor negative (POF-), and exhibit a high rate of fermentation, similar to previously characterized lineages of domesticated yeast. Additionally, kveik yeasts are reportedly high-temperature tolerant, likely due to the traditional practice of pitching yeast into warm (>28°C) wort. Here, we characterize kveik yeasts from 9 different Norwegian sources via PCR fingerprinting, whole genome sequencing of selected strains, phenotypic screens, and lab-scale fermentations. Phylogenetic analysis suggests that kveik yeasts form a distinct group among beer yeasts. Additionally, we identify a novel POF- loss-of-function mutation, as well as SNPs and CNVs potentially relevant to the thermotolerance, high ethanol tolerance, and high fermentation rate phenotypes of kveik strains. We also identify domestication markers related to flocculation in kveik. Taken together, the results suggest that Norwegian kveik yeasts are a genetically distinct group of domesticated beer yeasts with properties highly relevant to the brewing sector.

## Introduction

It is clear that human activity resulted in the domestication of *Saccharomyces cerevisiae* yeasts specifically adapted for beer production. Recently, it has been shown that present-day industrial beer yeasts have originated from a handful of domesticated ancestors, with one major clade, “Beer 1,” comprising the majority of German, British, and American ale yeasts, and another clade, “Beer 2,” which does not have geographic structure and are more closely related to wine yeasts (Gallone et al., 2016). In general, it appears that human selection of beer yeasts over the span of centuries has resulted in the evolution of mechanisms to: efficiently ferment wort sugars such as maltose and maltotriose via duplications of *MAL* genes; eliminate the production of phenolic off flavor (POF) by frequent nonsense mutations in the genes *PAD1* and *FDC1*, responsible for production of 4-vinylguaiacol (4-VG), thereby generating POF negative (POF-) strains, and; flocculate efficiently, thereby assisting in the downstream processing of the product (McMurrough et al., 1996; Brown et al., 2010; Steensels and Verstrepen, 2014; Gallone et al., 2016; Gonçalves et al., 2016).

Regardless of the region of origin, beer yeast was likely maintained and domesticated by reuse (repitching) as well as sharing amongst generations of brewers, resulting in many of the domesticated beer yeasts used in the present day (Gibson et al., 2007; Libkind et al., 2011; Steensels et al., 2014; Gallone et al., 2016). It must not be assumed, however, that the domestication of beer yeasts occurred solely within the confines of industrial breweries, as there were farmhouse brewing traditions predating the industrialization of beer across northern Europe (Nordland, 1969; Räsänen, 1975). These brewers used yeast strains they maintained themselves, and the same yeast was generally used for brewing and for baking. However, in Norway and Sweden, beer and unleavened breads predated leavened bread due to a lack of suitable grain (Visted and Stigum, 1971). Improvements in transportation and increasing economic specialization caused traditional farmhouse brewing to decline from the nineteenth century onwards, which coupled with the entry of commercial yeast likely led to the disappearance of many traditional brewing yeasts (Nordland, 1969).

**Figure 1:**
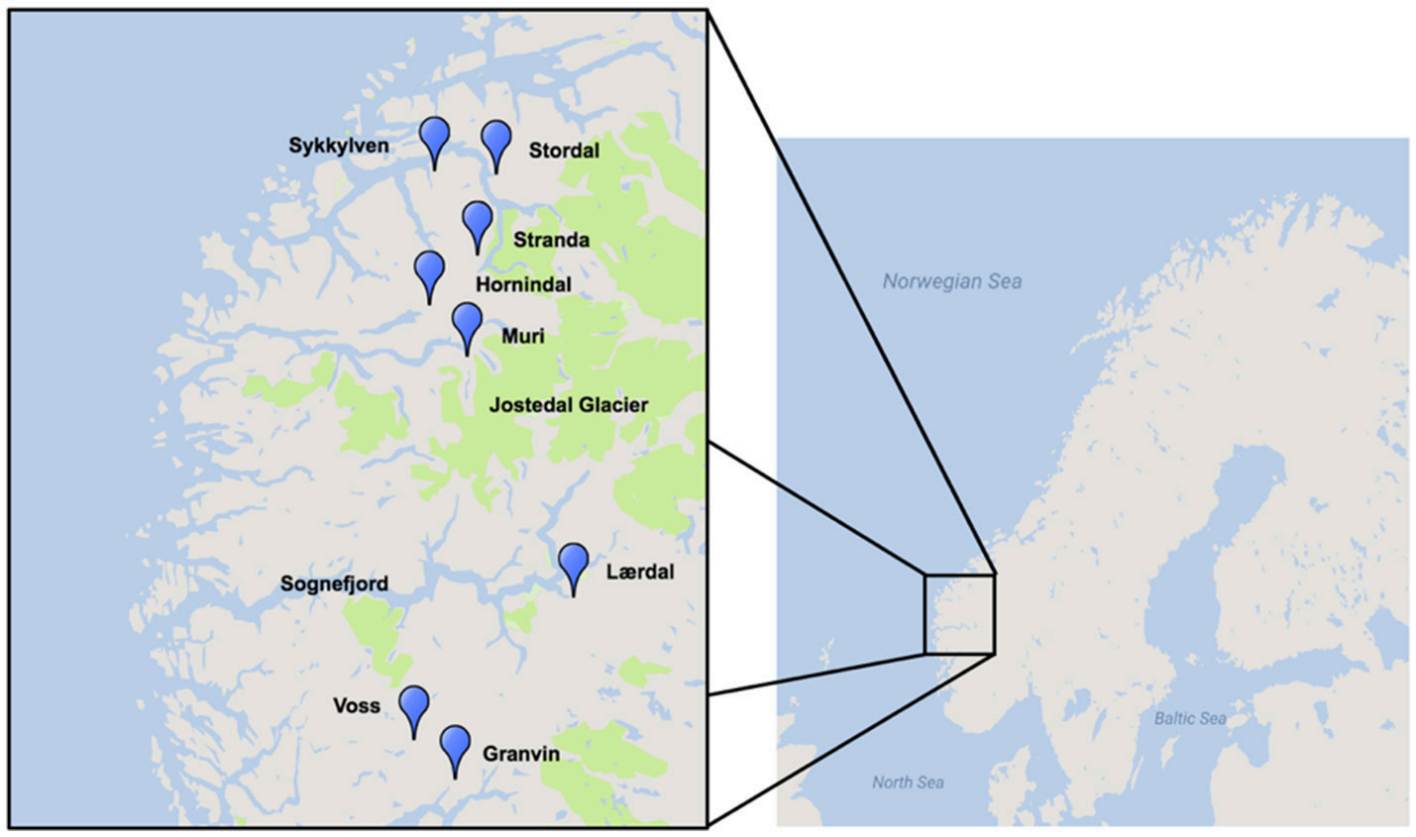
Geographical distribution of kveik yeast samples sourced for this project. Map was generated using Google Maps and Scribble Maps. Parks, including the Jostedalsbreen (Jostedal glacier) National Park are highlighted in green.

A region where traditional yeast cultures are still being used is western Norway, where a number of farmhouse brewers have maintained the traditional yeasts of this region, some reportedly for hundreds of years (Figure 1; Nordland, 1969). Norwegian farmhouse ale is produced predominantly from malted barley and is typically hopped, and also infused with juniper branches (Nordland, 1969). The farmhouse beers themselves are typically referred to as maltøl or kornøl. Until recently the yeast cultures, referred to as kveik, a dialect term for yeast in this region, were geographically isolated and maintained only locally by traditional farmhouse brewers. It is hypothesized that kveik yeasts are domesticated, as beers produced using these yeasts are reported to be non-phenolic (POF-) and these yeasts are potentially capable of rapidly fermenting malt-derived sugars due to the reported short fermentation times. Also, much like domesticated beer yeasts, kveik yeasts are maintained and reused via serial repitching (Gibson et al., 2007; Garshol, 2014; Stewart, 2015).

However, there are some critical differences in the way kveik is used and maintained that may have influenced its adaptive evolution and consequently impacted the generation of specific phenotypic characteristics. First, kveik has historically been stored dried for extended time periods of up to 1 year or more (Nordland, 1969). Second, kveik is typically inoculated by pitching into barley wort of between 28 and 40°C (Supplementary Table S1), a very high fermentation temperature for beer yeast (Caspeta and Nielsen, 2015). The most common temperature cited in older sources is “milkwarm,” meaning the temperature of milk as it leaves the udder, which is about 35°C (Iacobsen, 1935; Nordland, 1969; Strese and Tollin, 2015). Third, this wort is often of high sugar content (up to ~1.080 SG/19.25°Plato, compared to a typical wort of 1.050 SG or 12.5°Plato), and the brewers prefer a short fermentation time, often of only 1–2 days before transferring to a serving vessel (Nordland, 1969; Garshol, 2014). Traditionally, in the areas from which the studied yeast cultures come, the wort would be made from home-made barley malts, as barley was the main crop in these areas, and also the preferred grain for brewing (Hasund, 1942). The yeast is typically collected from the foam of the fermenting beer, or from the bottom slurry after primary fermentation, and dried until its next usage (Nordland, 1969). If the yeast went bad or was too old, the brewer would borrow yeast from neighbors, often choosing those who were known for having good beer (Nordland, 1969). Taken together, this adaptive environment for kveik yeasts was somewhat different from most industrial ale yeasts, while still favoring the possible development of domesticated traits.

Remarkably, yeast logs, specifically created for the storage of kveik, can be dated at least as far back as A.D. 1621 (Nordland, 1969), suggesting that kveik reuse began well before this date, as presumably the yeast was being reused prior to the development of specialized technology for yeast storage. This lines up with, and potentially predates, recent predictive modeling of the timeline of modern yeast domestication around A.D. 1573-1604 (Gallone et al., 2016). Kveik may therefore be a group of beer yeasts which have been domesticated and maintained by a geographically isolated brewing tradition, parallel to industrial beer production.

Yet, critically little is understood about kveik yeasts. While some of these yeasts have now been shared globally, there is a lack of empirical phenotypic and genotypic data pertaining to this intriguing group of beer yeasts. Here we report PCR fingerprinting and whole genome sequence data that suggest kveik yeasts form an interrelated group of beer yeasts genetically distinct from known domesticated beer yeasts. Our phenotypic characterizations and whole genome sequencing reveal evidence of domestication and positive characteristics in flavor compound production and stress tolerance that suggests the potential for kveik yeasts in a wide range of industrial applications.

## Materials and methods

### Yeast strains

A total of 9 samples of Norwegian kveik and one additional Lithuanian farmhouse ale yeast sample were analyzed in the study. Seven kveik were supplied as liquid slurries, and two were supplied as dried yeast samples. The dried samples were rehydrated in sterile water. The liquid yeast slurries were enriched by inoculating 50 μl of the slurry into 5 mL YPD (1% yeast extract; 2% peptone; 2% dextrose). The samples were incubated at 30°C for 24 h with shaking, then streak plated onto Wallerstein Nutrient agar (WLN; Thermo Fisher CM0309), a differential medium for yeasts that distinguishes multiple yeasts from each other within one sample on the basis of uptake of the bromocresol green dye. Yeast colonies were then substreaked onto WLN to ensure purity. The resultant strains are summarized in Table 1. Additional control strains for the experiments are listed in Table 1.

**Table 1:** Investigated yeast strains, source information, and sequence identification.

**Strain name**	**Source**	**GenBank Accession #**	**References**
Stordal Ebbegarden 1^†^	Jens Aage Øvrebust; Stordal, Norway	MG641161	This study
Stordal Ebbegarden 2	Jens Aage Øvrebust; Stordal, Norway	MG641162	This study
Stordal Framgarden 1	Petter B. Øvrebust; Stordal, Norway	MG641163	This study
Stordal Framgarden 2	Petter B. Øvrebust; Stordal, Norway	MG641164	This study
Granvin 1^†^	Hans Haugse; Granvin, Norway	MG641170	This study
Granvin 2	Hans Haugse; Granvin, Norway	MG641171	This study
Granvin 3	Hans Haugse; Granvin, Norway	MG754414	This study
Granvin 4	Hans Haugse; Granvin, Norway	MG709026	This study
Granvin 5	Hans Haugse; Granvin, Norway	MG709027	This study
Granvin 6	Hans Haugse; Granvin, Norway	MG709028	This study
Granvin 7	Hans Haugse; Granvin, Norway	MG709029	This study
Granvin 8	Hans Haugse; Granvin, Norway	MG709030	This study
Granvin 9	Hans Haugse; Granvin, Norway	MG709031	This study
Hornindal 1^†^	Terje Raftevold; Hornindal, Norway	MG641172	This study
Hornindal 2^†^	Terje Raftevold; Hornindal, Norway	MG641173	This study
Hornindal 3	Terje Raftevold; Hornindal, Norway	MG641174	This study
Joniškelis	Julius Simonaitis; Joniškelis, Lithuania	MG719970	This study
Lærdal 1	Dagfinn Wendelbo; Lærdal, Norway	MG641175	This study
Lærdal 2^†^	Dagfinn Wendelbo; Lærdal, Norway	MG641176	This study
Muri^*^	Bjarne Muri; Olden, Norway	MG641177	This study
Stranda	Stein Langlo; Stranda, Norway	MG641165	This study
Sykkylven 1	Sigurd Johan Saure; Sykkylven, Norway	MG641166	This study
Sykkylven 2	Sigurd Johan Saure; Sykkylven, Norway	MG641167	This study
Voss 1^†^	Sigmund Gjernes; Voss, Norway	MG641168	This study
Voss 2	Sigmund Gjernes; Voss, Norway	MG641169	This study
BBY002 (Vermont Ale)^†^	Escarpment Laboratories; Canada	–	This study
WLP001^†^	White Labs; USA	–	This study; Rogers et al. (2016)
WLP002	White Labs; USA	–	This study
WLP007	White Labs; USA	–	This study; Kopecká et al. (2016)
WLP029	White Labs; USA	–	This study
WLP090	White Labs; USA	–	This study
WLP570	White Labs; USA	–	This study; Kopecká et al. (2016)
WLP585	White Labs; USA	–	This study
WLP590	White Labs; USA	–	This study
WLP045	White Labs; USA	–	This study
WLP050	White Labs; USA	–	This study
WY1007	Wyeast; USA	–	This study
WY1272	Wyeast; USA	–	This study
WY1318	Wyeast; USA	–	This study
WY2575	Wyeast; USA	–	This study
RC212	Lallemand; Canada	–	This study
EC1118	Lallemand; Canada	–	This study; Novo et al. (2009)
Idun_1	Idun Industri; Norway	–	This study
Idun_2	Idun Industri; Norway	–	This study
K701	Brewing Society of Japan, Japan	–	This study; Watanabe et al. (2013)
WildThing	Escarpment Laboratories; Canada	–	This study

Sequence identification was performed via ITS1-ITS4 rDNA amplification, sequencing, and BLAST.

† Strains selected for whole genome sequencing are indicated.

* *Saccharomyces cerevisiae/eubayanus/uvarum. All other strains are Saccharomyces cerevisiae. Strain selected for whole genome sequence analysis*.

### DNA extraction

DNA was extracted using an adaptation of a previously described method (Ausubel et al., 2002). Briefly, yeast cells were grown in 3 mL of YPD broth at 30°C, 170 rpm for 24 h, washed with sterile water, and pelleted. The cells were resuspended in 200 μL of breaking buffer (2% Triton X-100, 1% SDS, 100 mM NaCl, 10 mM Tris-HCl). 0.3 g of glass beads and 200 μL of phenol/chloroform/isoamyl alcohol was added and the samples were vortexed continuously at maximum speed for 3 min to lyse the cells. Following centrifugation, the aqueous layer was transferred to a clean tube and 1 mL of 100% ethanol was added. The supernatant was removed following another centrifugation step. The resulting pellet was resuspended in 400 μL of 1X TE buffer and 30 μL of 1 mg/mL DNase-free RNase A and incubated at 37°C for 5 min. The pellet was then washed with 1 mL of 100% ethanol and 10 μL of 4 M ammonium acetate, followed by another wash with 1 mL of 70% ethanol, and then resuspended in 100 μL of sterile ddH_2_O.

### PCR and ITS sequencing

The internally transcribed spacer (ITS) regions of the yeast strains were amplified using ITS1 and ITS4 primers (Pham et al., 2011). PCR reactions contained 1 μL of genomic DNA, 2.5 μM of each primer, 0.4 mM dNTPs, 2.5 U of Taq DNA polymerase, and 1X Taq reaction buffer. The amplification reactions were carried out in a BioRad T100 Thermocycler under previously described conditions (Pham et al., 2011). PCR products were visualized on a 1% agarose gel in 1X TAE buffer to confirm successful amplification. The samples were purified using the QIAquick PCR purification kit and sequenced using an Applied Biosystems 3730 DNA analyzer. 4peaks software was used to perform quality control of sequence traces. The resulting sequences were analyzed for species-level homology using NCBI BLAST (blastn suite).

### DNA fingerprinting

Yeast strains were identified by interdelta PCR fingerprinting using interdelta primers δ2 (5′-GTGGATTTTTATTCCAACA-3′), δ12 (5′-TCAACAATGGAATCCCAAC-3′), and δ21 (5′-CATCTTAACACCGTATATGA-3′) (Ness et al., 1993; Legras and Karst, 2003). Primer pairs selected for further amplification and analysis were δ2 + δ12 and δ12 + δ21, which both yielded the greatest range of well-resolved bands. PCR was carried out as follows: 4 min at 95°C, then 35 cycles of 30 s at 95°C, 30 s at 46°C, then 90 s at 72°C, followed by a final 10 min step at 72°C (Legras and Karst, 2003). Reaction products were confirmed through electrophoresis on a 1% agarose gel in 1X TAE buffer. PCR samples were then purified using a QIAquick PCR purification kit and analyzed on an Agilent 2100 Bioanalyzer using the Agilent DNA 7500 chip. Banding patterns obtained using Bioanalyzer were analyzed using GelJ software (Heras et al., 2015). Comparisons for each primer set (δ2 + δ12 and δ12 + δ21) were generated independently using the Comparison feature of the software, clustering the fingerprints using Pearson correlation and UPGMA (Heras et al., 2015). Resultant individual distance matrices were combined using fuse.plot in R (https://github.com/andrewfletch/fuse.plot), which uses the hclust algorithm to format and fuse the matrices and perform hierarchical clustering with UPGMA. The data were visualized using FigTree software (http://tree.bio.ed.ac.uk/software/figtree/).

### DNA content by flow cytometry

Flow cytometry was performed on six kveik strains to estimate ploidy essentially as described by Haase and Reed (2002). Cells were grown overnight in YPD medium, and ~1 × 10^7^ cells were washed with 1 mL of 50 mM citrate buffer. Cells were then fixed with cold 70% ethanol, and incubated overnight at −20°C. Cells were then washed with 50 mM citrate buffer (pH 7.2), resuspended in 50 mM citrate buffer containing 0.25 mg mL^−1^ RNAse A and incubated overnight at 37°C. 1 mg mL^−1^ of Proteinase K was then added, and cells were incubated for 1 h at 50°C. Cells were then stained with SYTOX Green (2 μM; Life Technologies, USA), and their DNA content was determined using a FACSAria IIu cytometer (Becton–Dickinson, USA). DNA contents were estimated by comparing fluorescence intensities with those of *S. cerevisiae* haploid (CEN.PK113-1A) and diploid (CEN.PK) reference strains. One hundred thousand events were collected per sample during flow cytometry. Data was processed with the “flowCore” package (Hahne et al., 2009) in R, while mean peak fluorescence intensities were estimated with the “normalmixEM” function of the “mixtools” package (Benaglia et al., 2009) in R.

### Genome sequencing and analysis

The whole genomes of eight strains (six kveik strains and two commercial brewing strains as controls; see Table 1) were sequenced by Genome Québec (Montreal, Canada). In brief, DNA was isolated as described above, after which an Illumina TruSeq LT paired-end 150 bp library was prepared for each strain and sequencing was carried out with a HiSeqX instrument. Sequencing reads were quality-analyzed with FastQC (version 0.11.5) (Andrews, 2010) and trimmed and filtered with Trimmomatic (version 0.36; see Supplementary Table S2 for parameters) (Bolger et al., 2014). Reads were aligned to a *S. cerevisiae* S288c (R64-2-1) reference genome using SpeedSeq (0.1.0) (Chiang et al., 2015). Quality of alignments was assessed with QualiMap (2.2.1) (García-Alcalde et al., 2012). Variant analysis was performed on aligned reads using FreeBayes (1.1.0-46-g8d2b3a0l; see Supplementary Table S2 for parameters) (Garrison and Marth, 2012). Variants in all strains were called simultaneously (multi-sample). Prior to variant analysis, alignments were filtered to a minimum MAPQ of 50 with SAMtools (1.2; see Supplementary Table S2 for parameters) (Li et al., 2009). Annotation and effect prediction of the variants was performed with SnpEff (1.2; see Supplementary Table S2 for parameters) (Cingolani et al., 2012). Copy number variations of chromosomes and genes were estimated based on coverage with Control-FREEC (11.0; see Supplementary Table S2 for parameters) (Boeva et al., 2012). Statistically significant copy number variations were identified using the Wilcoxon Rank Sum test (*p* < 0.05). The median coverage and heterozygous SNP count over 10,000 bp windows was calculated with BEDTools (2.26.0) (Quinlan and Hall, 2010) and visualized in R.

### Phylogenetic and population structure analysis

Prior to phylogenetic and population structure analysis, consensus genotypes for the sequenced strains were called from the identified variants using BCFtools (1.2) (Li, 2011). Because of the high levels of heterozygosity (>50,000 heterozygous SNPs) in the six kveik strains, haplotype phasing was also attempted using WhatsHap (0.14.1) (Martin et al., 2016). WhatsHap is a read-based phasing tool, that uses mapped sequencing reads spanning at least two heterozygous variants to infer phase. The consensus haplotypes were called from the phased variants using BCFtools. Genome assemblies of the 157 *S. cerevisiae* strains described in Gallone et al. (2016) were retrieved from NCBI (BioProject PRJNA323691). In addition, the genome assembly of *Saccharomyces paradoxus* CBS432 was retrieved from https://yjx1217.github.io/Yeast_PacBio_2016/data/ (Yue et al., 2017) to be used as an outgroup. Multiple sequence alignment of the consensus genotypes of the eight sequenced strains and the 158 assemblies was performed with the NASP pipeline (1.0.0) (Roe et al., 2016) using *S. cerevisiae* S288c (R64-2-1) as the reference genome. A matrix of single nucleotide polymorphisms (SNPs) in the 167 strains was extracted from the aligned sequences. The SNPs were annotated with SnpEff (Cingolani et al., 2012) and filtered as follows: only sites that were in the coding sequence of genes, present in all 167 strains and with a minor allele frequency >1% (one strain) were retained. The filtered matrix contained 4161584 SNPs (142120 sites). A maximum likelihood phylogenetic tree was estimated using IQ-TREE (1.5.5; see Supplementary Table S2 for parameters) (Nguyen et al., 2015). IQ-TREE was run using the “GTR+F+R4” model and 1000 ultrafast bootstrap replicates (Minh et al., 2013). The resulting maximum likelihood tree was visualized in iTOL (Letunic and Bork, 2016) and rooted with *S. paradoxus* CBS432. The above steps from multiple sequence alignment onwards were repeated with the phased consensus haplotypes of the six kveik strains.

The population structure of 165 strains was investigated using the model-based algorithms in STRUCTURE (2.3.4; see Supplementary Table S2 for parameters) (Pritchard et al., 2000) and fastStructure (1.0; see Supplementary Table S2 for parameters) (Raj et al., 2014). Both tools were run on multiple threads using structure_threader (1.2.4; see Supplementary Table 2 for parameters) (Pina-Martins et al., 2017). The SNP matrix produced from the multiple sequence alignment was filtered using PLINK (1.9; see Supplementary Table S2 for parameters) (Purcell et al., 2007) by removing sites in linkage disequilibrium (using a 50 SNP window size, 5 SNP step size, and pairwise threshold of 0.5) and with a minor allele frequency <5%. In addition, SNPs from *S. cerevisiae* S288c and *S. paradoxus* CBS432 were excluded from the population structure analysis. The thinned SNP matrix, now consisting of 26583 sites, was used as input to both STRUCTURE and fastStructure, which were run for 1 to 11 ancestral populations (*K*). The SNP matrix is available as Supplementary Data Sheet 1. The STRUCTURE algorithm was run in 10 independent replicates for each *K* value and with an initial burn-in period of 100,000 iterations, followed by 100,000 iterations of sampling. The number of ancestral populations (*K*) that best represented this dataset was chosen based on the “Evanno method” (Evanno et al., 2005; Earl and vonHoldt, 2012) for the STRUCTURE results with STRUCTURE HARVESTER and by the *K* value that maximized marginal likelihood for the fastStructure results (Raj et al., 2014). The STRUCTURE results were finally clustered with the online CLUMPAK server (Kopelman et al., 2015). Results were plotted in “distruct”-type plots in R. Principal component analysis of the thinned SNP matrix produced for population structure analysis was also performed using the SNPRelate package (Zheng et al., 2012). Nucleotide diversities within and between populations were estimated in R using the PopGenome package (Pfeifer et al., 2014).

### Wort preparation

Wort used for beer fermentations and yeast propagation was obtained from a commercial brewery, Royal City Brewing (Guelph, ON). The hopped wort was prepared using Canadian 2-row malt to an original gravity of 12.5°Plato (1.050 specific gravity). The wort was sterilized prior to use at 121°C for 20 min, and cooled to the desired fermentation or propagation temperature overnight.

### Propagation and fermentation

Colonies from WLN plates were inoculated into 5 mL of YPD and grown at 30°C, 170 rpm for 24 h. The YPD cultures were transferred into 50 mL of sterilized wort and grown at 30°C, 170 rpm for 24 h. These cultures were counted using a haemocytometer and inoculated at a rate of 1.2 × 10^7^ cells/mL into 50 mL of sterilized wort in glass “spice jars” (glass jars of total volume 100 mL with straight sides) fitted with airlocks. These small-scale fermentations were performed in triplicate at 30°C for 12 days. 30°C was chosen as the fermentation temperature as it is a common temperature in Norwegian farmhouse brewing (Supplementary Table S1). The jars were incubated without shaking to best approximate typical beer fermentation conditions. Fermentation profiles were acquired by weighing the spice jars to measure weight loss, normalizing against water evaporation from the airlocks.

### Beer metabolite analysis

Following fermentation, samples were collected and filtered with 0.45 μm syringe filters prior to metabolite analysis. Flavor metabolite analysis was performed using HS-SPME-GC-MS (Rodriguez-Bencomo et al., 2012). Samples contained 2 mL of beer, 0.6 g of NaCl, 10 μL of 3-octanol (0.01 mg/mL), and 10 μL of 3,4-dimethylphenol (0.4 mg/mL). 3-octanol and 3,4-dimethylphenol were used as internal standards. The ethanol and sugar content was measured using HPLC and a refractive index (RI) detector. The samples were analyzed using an Aminex HPX-87H column, using 5 mM sulfuric acid as the mobile phase, under the following conditions: flow rate of 0.6 mL/min, 620 psi, and 60°C. Each sample contained 400 μL of filtered beer and 50 μL of 6% (v/v) isopropanol as the internal standard.

### Phenotypic assays

To determine temperature tolerance, yeast grown for 24 h at 170 rpm at 30°C in YPD were subcultured into YPD pre-warmed to specified temperatures (30, 40, 42, 43, 45°C) in duplicate to an initial OD_600_ of 0.1 and incubated with shaking for 20 h at the indicated temperature. To determine ethanol tolerance, yeast cultures grown for 24 h at 170 rpm at 30°C in YPD were sub-cultured into YPD containing increasing concentrations of ethanol (YPD + EtOH 10, 12, 14, 15, 16%) in duplicate to an initial OD_600_ of 0.1 and incubated with shaking for 20 h at the indicated temperature. To assess growth yield for temperature tolerance and ethanol tolerance, the yeast samples were subjected to declumping using phosphoric acid and immediate OD_600_ measurements were taken using a spectrophotometer (Simpson and Hammond, 1989). To determine flocculation, yeast cultures were grown for 24 h at 170 rpm at 30°C in YPD, and then 0.5 mL was inoculated into 5 mL sterilized wort, which was incubated for 24 h at 170 rpm at 30°C. Flocculation was assessed using the spectrophotometric absorbance methodology of ASBC method Yeast-11 (ASBC, 2011). Values are expressed as % flocculance, with <20% representing non-flocculant yeast and >85% representing highly flocculant yeast.

### Statistical analysis

Statistical analysis was performed on the fermentation, metabolite and phenotypic data with one-way ANOVA and Tukey's test using the “agricolae” package in R (http://www.r-project.org/). The results of the statistical tests are available as Supplementary Data Sheet 2.

## Results

### Kveik are a genetically distinct group of beer yeasts

In order to determine whether original kveik samples contain multiple yeast strains, the kveik samples were first plated on WLN agar, which is a differential medium allowing for distinguishing of *Saccharomyces* on the basis of differences in colony morphology and uptake of the bromocresol green dye (Hutzler et al., 2015). We found that all but two of the kveik samples contained more than one distinct yeast colony morphology, corresponding to potentially unique strains. The number of strains isolated from individual kveik cultures thus ranged from 1 to 9 and totaled 25 and is summarized in Table 1.

Given that anecdotal reports stated kveik yeasts are often flocculent, demonstrate a fast fermentation rate, and are capable of utilizing malt sugars, all of which are hallmarks of domestication (Gallone et al., 2016), we aimed to determine the closest likely relatives of kveik yeasts among known strains of *S. cerevisiae*, and to determine whether kveik yeasts are related to each other. As nearly all domesticated ale yeasts belong to the *S. cerevisiae* species, we hypothesized that the kveik isolates also belong to *S. cerevisiae* (Almeida et al., 2015; Gallone et al., 2016; Gonçalves et al., 2016). We performed ITS sequencing and found that all but one kveik strain was identified (via BLAST search) as *S. cerevisiae* (Table 1). We found that the strain originating from Muri is most closely homologous to previously identified *S. cerevisiae/eubayanus/uvarum* triple hybrids, presenting this particular yeast strain as an intriguing potential domesticated hybrid warranting further investigation (Table 1).

Since the kveik yeasts appear to be *S. cevevisiae* strains, we next asked how they relate genetically to other *S. cerevisiae* yeasts. In order to answer this question, we performed interdelta PCR using the δ12/21 and δ2/12 primer sets (Legras and Karst, 2003; Hutzler et al., 2015). The δ elements are separated by amplifiable distances in the *S. cerevisiae* genome, and consequently interdelta PCR can be used to amplify interdelta regions, which in turn can be used to rapidly fingerprint yeasts for comparative genetic purposes (Legras and Karst, 2003; Hutzler et al., 2015).

Preliminary trials using the δ1/2, δ2/12, and δ12/21 primer sets showed that the latter two primer sets produced the greatest range of useful bands when separated via agarose gel electrophoresis. We then amplified the δ2/12 and δ12/21 regions of all the kveik strains and a selection of yeast strains representing “Beer 1” (German, American, UK), “Beer 2” (Belgian Saison), saké, wine, bread, wild, and distilling yeasts. Separation was performed using capillary gel electrophoresis (Agilent Bioanalyzer), which yielded greater accuracy and sensitivity (Hutzler et al., 2015). Analysis of both δ2/12 and δ12/21 datasets individually revealed that the kveik yeasts formed a subgroup among the other domesticated yeasts, such that the kveik yeasts appeared to be more closely related to each other than to other domesticated yeasts (Supplementary Figure S1). We next created a composite analysis of the interdelta datasets, yielding a dendrogram which placed some beer strains close together (Supplementary Figure S2). We found that a group of strains from German, British and American origin (WLP029, WLP002, WY1272, WLP007, BBY002) were represented in the dendrogram, and may represent the “Beer 1” clade (Belgian/German, British, American), as identified by Gallone et al. (2016). However, the kveik yeasts formed a group of related yeasts with a likely common ancestor. The kveik yeasts seem to be related to the beer strains more closely than other yeast groups. Furthermore, other yeasts from this study such as the hybrid Muri yeast, a Norwegian bread yeast (Idun) and the Lithuanian yeast strain (Joniškelis) do not appear to fit within the kveik family. Taken together these results suggest that kveik yeasts could represent a genetically distinct group of yeasts. While it does not properly resolve phylogeny due to lack of detail, the interdelta fingerprinting method can be used to assess which kveik yeasts are closely related to each other, and which could be selected for further sequencing analysis such that a representative range of strains are selected.

In order to better understand the genomics of kveik in relation to other *S. cerevisiae* yeasts, the whole genomes of six kveik strains (Table 1) were sequenced using 150 bp paired-end Illumina technology to an average coverage ranging from 472 × to 1,221 × (Table 2). These strains were selected based on the DNA fingerprinting results to represent different subgroups of the kveik family. In addition, two control strains (WLP001 and Vermont Ale) were sequenced and included in the phylogenetic analysis. Flow cytometry and allele frequency distributions suggested that all six kveik strains were tetraploid (Table 2, Supplementary Figures S3–S5). However, 4/6 strains did show aneuploidy due to chromosomal CNVs, and of particular note, 3/6 strains containing an additional copy of chromosome IX. The kveik strains also showed high levels of heterozygosity, as the number of heterozygous SNPs ranged from ~54,000 to 68,000 (Table 2). The heterozygous SNP density was relatively uniform in the strains, with few regions having undergone loss of heterozygosity (Supplementary Figure S6).

**Table 2:** Estimated ploidy, spore viability, mean sequencing coverage along *S. cerevisiae* S288c reference genome, and number of heterozygous single nucleotide polymorphisms (SNPs) in the six sequenced kveik strains.

**Strain**	**Estimated ploidy**	**Spore viability (%)**	**Sequencing coverage (×)**	**Heterozygous SNPs**
Granvin 1	3.93 (±0.30)	56.5	946	65835
Hornindal 1	3.82 (±0.29)	59.0	1221	67910
Hornindal 2	4.10 (±0.23)	53.3	974	61402
Laerdal 2	4.03 (±0.22)	40.6	472	59090
Stordal Ebbegarden 1	3.92 (±0.23)	5.9	671	54344
Voss 1	3.88 (±0.26)	63.4	1198	64959

**Figure 2:**
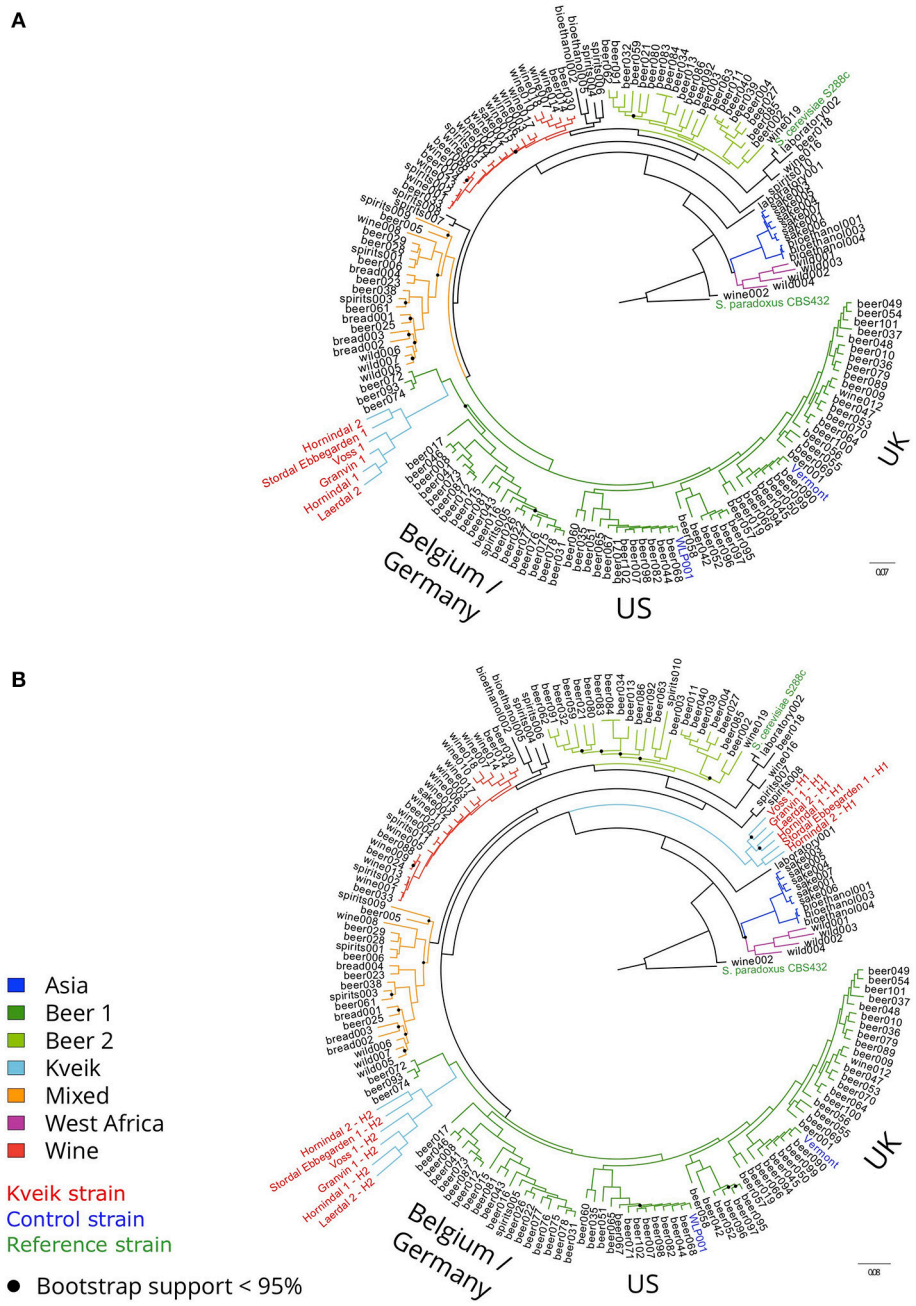
Phylogeny of the six sequenced kveik strains compared with two control strains and the 157 *S. cerevisiae* strains sequenced in Gallone et al. (2016). **(A)** Maximum likelihood phylogenetic tree based on SNPs at 142120 sites in 166 *S. cerevisiae* strains (rooted with *S. paradoxus* as outgroup). Black dots on nodes indicate bootstrap support values <95%. Branches are colored according to lineage, and strain names are colored according to type (kveik, red; control, blue; reference, green). Branch lengths represent the number of substitutions per site. **(B)** Maximum likelihood phylogenetic tree produced as in **(A)**, but using the phased haplotypes of the kveik yeasts instead of their consensus genotypes.

To examine the genetic relationship between kveik and other domesticated *S. cerevisiae* strains, phylogenetic and population structure analyses were performed together with genome sequences published elsewhere. First, the genome assemblies of the 157 *S. cerevisiae* strains investigated by Gallone et al. (2016) were retrieved from NCBI (PRJNA323691), while consensus genotypes of the six kveik and two control strains were produced from the SNPs and short InDels that were identified. After multiple sequence alignment and SNP identification, a filtered matrix containing 4161584 SNPs across 142120 sites was obtained (the SNP matrix is available as Supplementary Data Sheet 1). A maximum-likelihood phylogenetic tree was inferred from these polymorphic sites (Figure 2A). The main lineages reported in the original study (Gallone et al., 2016) were successfully reconstructed, and the two control strains clustered in the correct groups (“WLP001” in the “Beer 1–US” group, and “Vermont Ale” in the “Beer 1–UK” group). Consistent with the DNA fingerprinting results, the six kveik strains formed their own subgroup within the “Beer 1” group and appeared genetically distinct from other brewing yeasts, but closest to a group of German wheat beer yeasts known to contain mosaic genomes (beer072, 074, 093). To ensure that the high levels of heterozygosity in the six kveik strains wouldn't skew the results, read-based phasing of the kveik strain haplotypes was also performed. The analysis was repeated for the two phased haplotypes (Figure 2B), and the phylogeny revealed that one haplotype again formed a subgroup within the “Beer 1” group, while the other haplotype formed a unique group between the “Asia” and “Mixed” groups. This is suggestive of a hybrid origin for kveik consisting of both a Beer 1 and an unknown lineage. However, Illumina paired-end data is not ideal for read-based phasing, as many pairs of heterozygous SNPs might not be connected by a read pair. Long read sequencing, e.g., using PacBio or Nanopore technology, could be used to improve the quality and length of the haplotype blocks (Martin et al., 2016). This in turn would allow for a more detailed analysis of the ancestry of the kveik strains.

**Figure 3:**
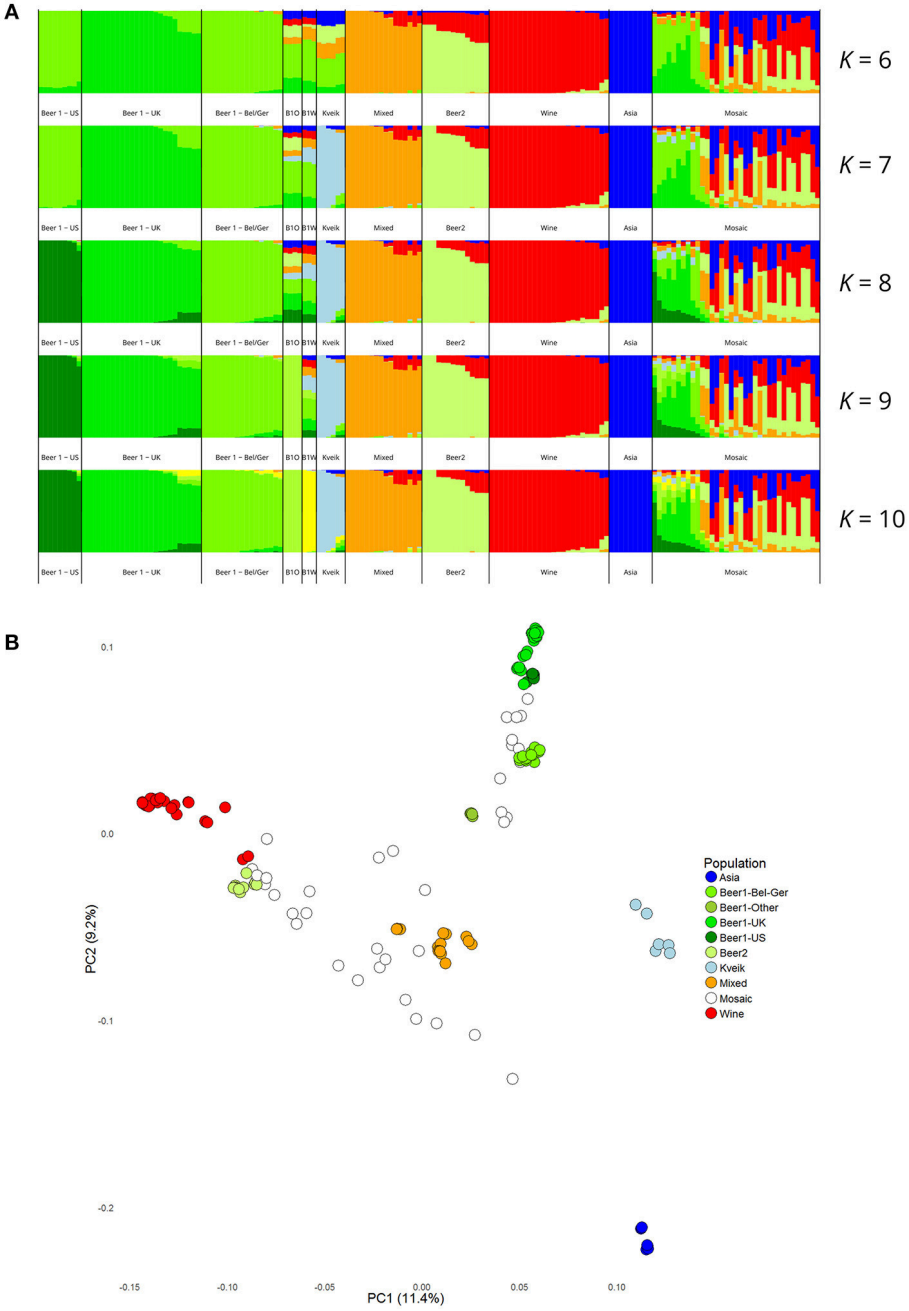
Population structure of the six sequenced kveik strains and the 157 *S. cerevisiae* strains sequenced in Gallone et al. (2016). **(A)** Population structure of 163 *S. cerevisiae* strains estimated with *STRUCTURE* based on SNPs at 26583 sites. Each strain along the x-axis is represented by a vertical bar partitioned into colors based on estimated membership fractions to the resolved populations for *K* = 6, 7, 8, 9, and 10 assumed ancestral populations. *K* = 9 best explains the data structure according to the “Evanno” method (Evanno et al., 2005). B1O: Beer 1–Other. **(B)** Principal component analysis of SNPs at 26583 sites in 163 *S. cerevisiae* strains. Dots are colored by population.

Population structure analysis was also performed based on the polymorphic sites among the 165 strains. First, the SNP matrix was filtered to remove sites in linkage disequilibrium and with minor allele frequencies <5%. The clustering algorithms STRUCTURE and fastStructure were then used on the thinned SNP matrix (26583 sites), and the resulting population structure was in agreement with the estimated phylogeny. The number of populations that best represented this dataset was nine (*K* = 9) for STRUCTURE (Figure 3A) and ten (*K* = 10) for fastStructure (Supplementary Figure S7). In both cases, the six kveik strains formed their own unique population, while the main populations reported in the Gallone et al. (2016) study were recreated. Even when the number of ancestral populations (*K*) was lowered to 7 or 8, the six kveik strains still formed a unique population (Figure 3A, Supplementary Figure S7A). The fastStructure analysis was also repeated to include the phased haplotypes (Supplementary Figure S7B), which revealed an admixed ancestry (with contributions from Asia, Beer 1, Mixed, and Wine populations) for one haplotype (H1), and placed the other haplotype (H2) in a population with outliers in the “Beer 1” lineage (beer015, 052, 095-097). The kveik haplotypes appear distinct from the German wheat beer yeasts, and the apparent connection of kveik to these yeasts suggested by the phylogeny is likely a coincidental artifact of both strain groups being mosaic/hybrid in origin. To support the population structure analysis, principal component analysis was performed on the thinned SNP matrix, which again clustered the six kveik strains separately from the other strains (Figure 3B). The per-site nucleotide divergence between the kveik population and the other populations was also higher than those observed between the other beer populations (Supplementary Table S3). As suggested by the DNA fingerprinting results, compared to the other beer populations, relatively high nucleotide diversity was also observed within the kveik population (Supplementary Table S4). Taken together, the results of the phylogenetic and population structure analysis suggest that the kveik strains selected for whole genome sequencing are genetically distinct from other domesticated yeasts.

### Brewing characteristics, domestication, and sporulation potential in kveik

We next sought to analyze the brewing-relevant parameters of kveik yeasts in pure culture fermentation. Since Norwegian kveik cultures appear to often contain multiple yeast strains, there is the possibility that strains are interdependent. It is therefore important to determine the fermentation characteristics of individual strains as single culture fermentations would show whether individual kveik strains can adequately ferment beer. An inability to do so would suggest there is an adapted advantage to the multi-strain nature of kveik cultures. Additionally, we aimed to confirm anecdotal reports that these yeasts exhibit short lag phases and display good fermentation kinetics.

**Figure 4:**
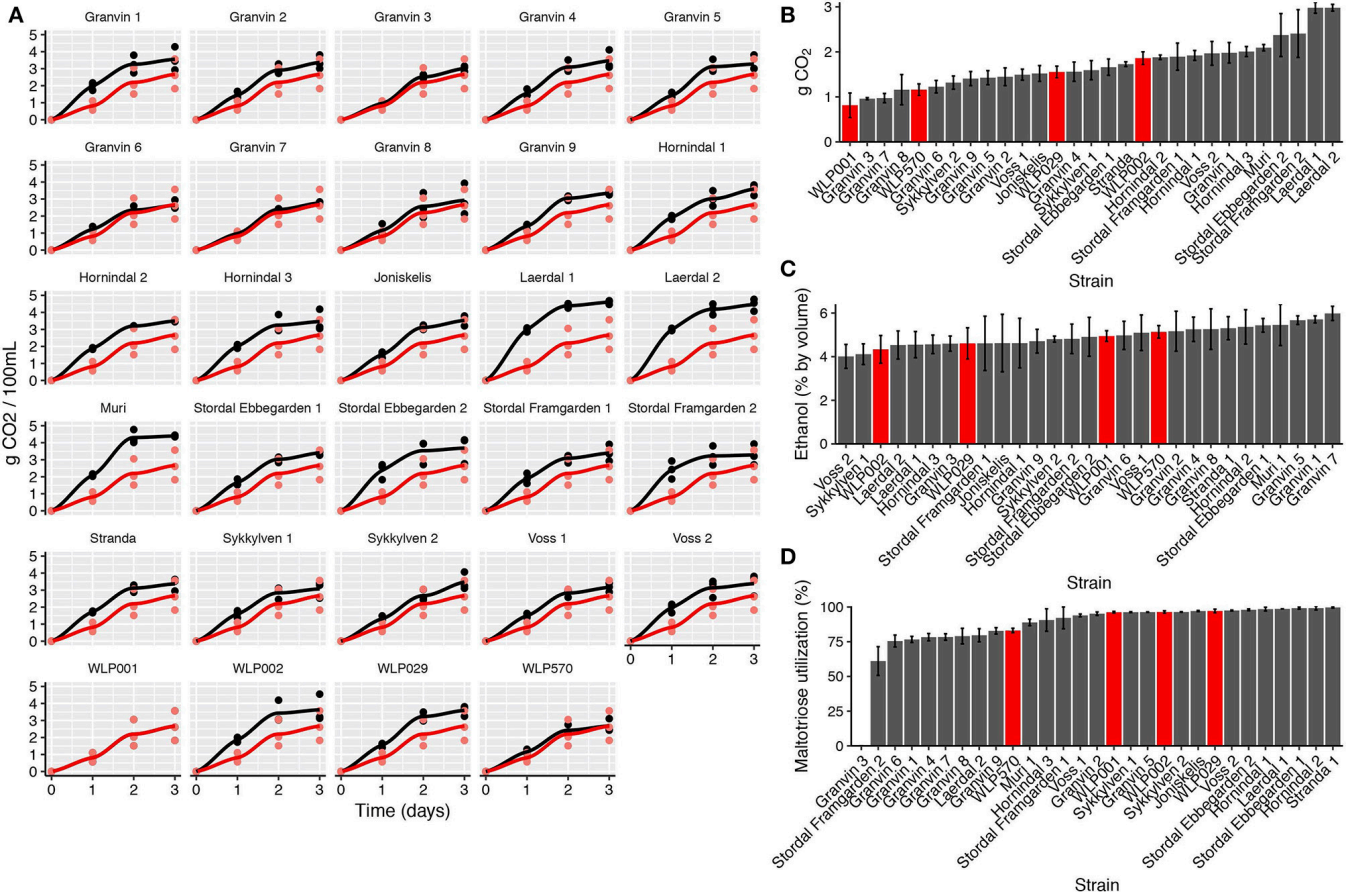
Fermentation kinetics and terminal ethanol concentration of small-scale wort fermentation (12.5°P original density) at 30°C. **(A)** CO_2_ evolution in the fermentations was calculated by weighing the fermentation vessels (50 mL) and normalizing for mass loss in the fermentation airlocks. The data were then multiplied to represent a 100 mL volume. Yeast strains (black) are compared to a control ale strain (WLP001; red). The first 3 days of fermentation are shown. **(B)** CO_2_ evolution at 24 h, calculated as in **(A)**. Control ale strains are marked in red. Error bars represent SD, *n* = 3. **(C)** Ethanol concentration was measured via HPLC following 12 days of fermentation. Error bars represent SD, *n* = 3. Control ale strains are marked in red. **(D)** Maltotriose utilization as calculated from residual maltotriose values and original maltotriose values of the wort. Control ale strains are marked in red.

We performed test fermentations using the pure culture kveik strains as well as relevant industrial ale yeast controls (WLP001, WLP002, WLP029, WLP570; White Labs). In particular, WLP001 was chosen because it is one of the most popular ale strains for craft beer production. The fermentations were performed at 30°C which has been reported to be a typical temperature for beers fermented using kveik (Garshol, 2015). In order to assess the fermentation rate during the early phases of wort fermentation, we monitored the CO_2_ loss in the fermentations via weighing. Using this technique, we observed that the fermentation curves for kveik was often favorable in comparison to the control strain with a shorter fermentation lag time observed in some of the strains (Figures 4A,B). Of the control strains, WLP002 produced the most CO_2_ after 24 h. We found that 11 of the kveik strains outperformed WLP002 at 24 h, with the best-performing strain (Laerdal 2) producing 70.6% more CO_2_ within the first 24 h of fermentation (Figure 4B). One-way ANOVA with Tukey's *post-hoc* test was performed and both Laerdal 1 and Laerdal 2 strains were determined to be significantly faster in this period at *P* < 0.05 (Supplementary Data Sheet 2). Following the 12-day fermentation and maturation period, we also measured the ethanol concentration of the beers using HPLC. The control ale strains produced ethanol values in the expected ascending order: WLP002 (4.33 ± 0.64%), WLP029 (4.60 ± 0.72%), WLP001 (4.94 ±0.25%), WLP570 (5.14 ± 0.29%). We found that the kveik yeasts produced expected ethanol yields within the expected range for beer strains of *S. cerevisiae*, with apparent attenuation ranges spanning 60–90%, and ethanol yield ranging from 4.01 ± 0.55 to 5.98 ± 0.32% (Figure 4C). Statistically significant groupings among the ethanol data were not observed (Supplementary Data Sheet 2). The control data combined with the ethanol yield from the kveik yeasts in wort fermentation indicates that the kveik yeasts attenuate wort within the expected range of industrial domesticated ale strains.

Domesticated brewing yeasts are characterized by their ability to efficiently use maltose and maltotriose (Gallone et al., 2016; Gonçalves et al., 2016). These sugars constitute the majority of the fermentable sugars in brewer's wort. As has been observed previously in brewing strains (Gallone et al., 2016; Gonçalves et al., 2016), the six sequenced kveik strains showed considerable copy number variations in genes related to maltose and maltotriose transport (Table 3). Significant amplifications in the entire *MAL3x* locus (containing the *MAL31* permease, *MAL32* maltase and *MAL33* transcription factor) and the putative maltose-responsive transcription factor *YPR196W* were observed in particular. Indeed, we also observed maltotriose utilization across the kveik strains in the wort test fermentations, with exception to the Granvin 3 strain (Figure 4D).

**Table 3:** Estimated copy numbers of genes linked to maltose transport in the six sequenced kveik strains.

**Strain**	***MAL1x***	***MAL3x***	***IMA2***	***MPH2***	***MPH3***	***YPR196W***
Granvin 1	2	14	5	1	4	10
Hornindal 1	5	14	5	1	4	11
Hornindal 2	6	14	6	1	4	13
Laerdal 2	4	11	6	1	4	9
Stordal Ebbegarden 1	5	11	6	0	4	14
Voss 1	2	17	7	0	4	15

To understand beer flavor contributions by the kveik yeasts, we also analyzed volatile aromatic compounds using HS-SPME-GC-MS (Table 4). Intriguingly, we found that all kveik yeasts belonging to the main kveik genetic lineage (Figure 2, Supplementary Figures 1, 2) produced minimal levels of 4-vinylguaiacol (clove, smoke), suggesting that the kveik family are POF- (Table 4). Indeed, these levels were significantly different from the POF+ control strain (WLP570) in all but one kveik yeast (Muri kveik; Supplementary Data Sheet 2). Non-domesticated *S. cerevisiae* strains tend to have functional *PAD1* and *FDC1* genes, allowing them to decarboxylate hydroxycinnamic acids to vinylphenols (Mukai et al., 2010). Many brewing strains lack the ability to produce such off-flavors, and studies have shown that these strains carry loss-of-function mutations in either *PAD1* and *FDC1* (Mukai et al., 2014; Gallone et al., 2016; Gonçalves et al., 2016). The six kveik strains sequenced here indeed carry loss-of-function mutations in these two genes (Table 5). Three of these mutations, 305G>A in *PAD1*, 460C>T in *FDC1*, and 501insA in *FDC1*, have been observed previously in brewing strains (Mukai et al., 2014; Gallone et al., 2016; Gonçalves et al., 2016), and are widespread among the strains belonging to the “Beer 1” population (Gallone et al., 2016). Notably, a 232A>T mutation in *FDC1*, causing a premature stop codon at position 78, was also observed in the Stordal Ebbegarden 1 strain. To our knowledge, this loss-of-function mutation in *FDC1* has not been reported before.

**Table 4:** Fermentation flavor metabolites (ppm) produced by kveik yeasts during wort fermentation at 30°C measured using HS-SPME-GC-MS.

	**Ethyl** **Acetate**	**Ethyl** **Caproate**	**Ethyl** **Caprylate**	**Ethyl Decanoate**	**Ethyl** **Nonanoate**	**Hexanoic** **Acid**	**Isoamyl** **Acetate**	**Isoamyl Alcohol**	**Isobutanol**	**Phenethyl** **Acetate**	**Phenethyl** **Alcohol**	**4-Vinyl** **Guaiacol**
Granvin 1	1.715	0.156	2.512	0.494	0.161	0.023	0.674	6.79	1.324	1.052	19.694	0.058
Granvin 2	3.118	0.366	4.555	0.455	0.197	0.01	0.781	7.879	1.527	1.87	21.603	0.012
Granvin 3	1.492	0.122	1.159	0.013	0.143	0.002	0.744	7.506	2.282	0.36	17.216	0.014
Granvin 4	1.195	0.059	0.232	0.012	0.025	0.004	0.467	4.719	1.126	0.257	15.163	0.043
Granvin 5	2.231	0.116	1.666	0.08	0.149	0.008	0.933	9.432	2.175	0.749	28.262	0.016
Granvin 6	3.2	0.365	5.005	0.88	0.238	0.02	0.905	9.046	1.9	1.36	24.966	0.016
Granvin 7	1.564	0.128	1.712	0.056	0.155	0.001	0.7	7.049	2.022	0.424	20.577	0.012
Granvin 8	1.229	0.056	0.299	0.026	0.028	0.003	0.538	5.423	1.344	0.298	14.628	0.043
Granvin 9	1.537	0.085	1.188	0.076	0.109	0.003	0.467	4.704	1.065	0.474	13.653	0.037
Hornindal 1	3.408	0.193	3.58	1.39	0.164	0.074	0.539	5.436	0.945	2.074	14.128	0.043
Hornindal 2	2.257	0.084	1.271	0.247	0.091	0.002	0.635	6.421	1.184	0.906	15.291	0.043
Hornindal 3	2.505	0.236	4.151	1.412	0.155	0.203^*^	0.556	5.659	0.838^*^	1.498	13.504	0.042
Joniskelis	1.495	0.117	2.301	1.277	0.151	0.055	0.589	5.942	1.018	1.568	17.63	0.223
Laerdal 1	1.838	0.315	4.124	0.891	0.204	0.116	0.453	4.689	0.624^*^	0.687	13.535	0.069
Laerdal 2	1.849	0.102	1.8	0.554	0.159	0.022	0.672	6.927	1.005	1.04	15.838	0.044
Muri	2.713	0.224	2.005	1.078	0.188	0.011	0.53	5.354	0.892	2.276	14.804	0.31
Stordal Ebbegarden 1	2.103	0.083	0.811	0.272	0.053	0.097	0.475	4.783	0.947	0.794	13.974	0.039
Stordal Ebbegarden 2	2.542	0.089	0.619	0.341	0.041	0.217^*^	0.677	7.052	1.135	1.074	16.637	0.049
Stordal Framgarden 1	2.395	0.168	2.975	0.772	0.158	0.058	0.55	5.536	0.901	1.635	15.809	0.052
Stordal Framgarden 2	2.654	0.44	4.112	0.753	0.176	0.006	0.593	5.998	0.976	0.864	14.03	0.047
Stranda	2.393	0.168	2.818	1.035	0.157	0.027	0.602	6.086	0.857	1.018	16.056	0.049
Sykkylven 1	2.046	0.101	1.306	0.427	0.08	0.005	0.483	4.883	0.867	0.749	14.28	0.043
Sykkylven 2	1.668	0.102	1.392	0.675	0.079	0.133	0.422	4.257	0.619^*^	0.622	12.081	0.044
Voss 1	2.156	0.209	3.317	0.618	0.145	0.006	0.463	4.651	0.941	0.825	12.377	0.039
Voss 2	2.364	0.307	3.059	0.347	0.157	0.005	0.519	5.225	1.01	1.148	15.121	0.039
WLP001	2.064	0.192	0.241	0.105	0.196	0.03	0.66	6.654	2.46	1.004	25.918	0.072
WLP002	0.735	0.076	0.537	0.047	0.101	0	0.81	8.168	4.062	0.478	19.481	0.053
WLP029	3.22	0.348	4.142	0.99	0.292	0.002	0.655	6.601	1.962	1.601	21.047	0.013
WLP570	5.734	0.806	8.586	1.583	0.424	0.019	1.395	14.057	2.106	3.529	33.427	0.299
Threshold (ppm)	30	0.21	0.9	0.2	0.85	8	1.2	70	100	3.8	100	0.3

*Fermentations were performed in triplicate. Metabolite values are shaded if present in quantities at or above above the stated sensory threshold values. Values presented are as mean ppm. Statistical analysis is available via Supplementary Data Sheet 2. Values marked with an asterisk are significantly different from all controls (P < 0.05, one-way ANOVA with Tukey's post-hoc test)*.

**Table 5:** Loss-of-function single nucleotide polymorphisms in *PAD1* and *FDC1* in the six sequenced kveik strains.

**Strain**	***PAD1***	***FDC1***		
	**305G>A**	**232A>T**	**460C>T**	**501insA**
	Trp102^*^	Lys78^*^	Gln154^*^	**Trp168fs**
Granvin 1	0/0/0/1		0/0/0/1	1/1/1/1
Hornindal 1	1/1/1/1		1/1/1/1	1/1/1/1
Hornindal 2	1/1/1/1		1/1/1/1	1/1/1/1
Laerdal 2	0/1/1/1		0/0/0/1	1/1/1/1
Stordal Ebbegarden 1	0/0/0/1	0/1/1/1	0/0/0/1	0/0/0/1
Voss 1	1/1/1/1		1/1/1/1	1/1/1/1

* *premature stop codon; ins, insertion; fs, frameshift*.

Also, analysis of the volatile ester profiles revealed the kveik yeasts produced above-threshold concentrations of three yeast fatty acid esters: ethyl caproate (pineapple, tropical; threshold 0.21 ppm), ethyl caprylate (tropical, apple, cognac; threshold 0.9 ppm), and ethyl decanoate (apple; threshold 0.2 ppm) (Engan, 1972; Meilgaard, 1982; Verstrepen et al., 2003; Comuzzo et al., 2006). However, significant differences were not observed in the concentrations of these esters relative to the various control strains. Isoamyl acetate (banana; threshold 1.2 ppm) was detected above threshold and significantly higher in WLP570 only (Supplementary Data Sheet 2), indicating that this is not a major ester component in the flavor profile of the kveik yeasts, or for the other industrial beer strains. Interestingly, loss-of-function mutations were identified in acetate ester-relevant genes *ATF1* and *ATF2* among 4/6 of the sequenced kveik strains (Supplementary Table S5). However, only one of these mutations was homozygous (“Laerdal 2”; homozygous *ATF1* lost stop codon) and was not linked to lower acetate ester formation in the beer fermentations (Table 4). Additionally, isobutanol levels were significantly lower among 3 kveik yeasts in comparison to the control ale strains, suggesting kveik may be capable of lower fusel alcohol production (Table 4, Supplementary Data Sheet 2).

We also analyzed the spore viability of the 6 sequenced kveik yeasts. Reasonable spore viability (40.6–63.4%) was observed in 5/6 of the strains, with one strain (“Stordal Ebbegarden 1”) showing low spore viability (Table 2). Interestingly, all sequenced kveik strains contain a loss of function mutation in *RMR1*, a protein required for meiotic recombination (Jordan et al., 2007). This mutation (726A>T causing lost stop codon) is only homozygous in the “Stordal Ebbegarden 1” strain, which may explain why this strain demonstrated low spore viability.

### Thermotolerance, ethanol tolerance, and flocculation in kveik

Since the initial fermentation trials demonstrated kveik yeasts are largely POF- and produce desirable fruity ester flavors, we next investigated the stress tolerance and flocculation of these yeasts to better determine their potential utility and to confirm these additional hallmarks of domestication. Given the reports of high-temperature fermentation by traditional Norwegian brewers (Nordland, 1969; Garshol, 2014), we monitored the growth of the kveik yeasts alongside known ale yeasts as control strains (WLP001; American ale, WLP029; German ale, WLP570; Belgian ale, WLP002; British ale) under normal and high temperature growth conditions (30–45°C).

We found that 19/25 kveik strains grew to >1.0 OD_600_ at 40°C, while only 1/4 of the control ale strains (WLP570) grew to this optical density at 40°C (Table 6). Furthermore, 11/25 kveik strains grew to >0.4 OD_600_ at 42°C, while only one of the control ale strains (WLP570) was able to. Remarkably, 19/25 kveik strains at least doubled its cell density at 43°C with the maximal optical density at this temperature observed for Laerdal 1 (OD_600_ 0.44). Interestingly, one of the control strains (WLP570) also showed growth at 43°C (OD_600_ 0.39). These data indicate that high temperature tolerance is common among kveik yeasts, and that high temperature tolerance is often limited among the American/British/German ale strains (Gallone et al., 2016). Notably, kveik strains displayed some growth up to 43°C, nearing the theoretical limit, and current technological upper threshold for *S. cerevisiae* cell growth (Caspeta et al., 2013, 2016; Caspeta and Nielsen, 2015). All strains failed to grow at 45°C (data not shown). A number of mutations in yeast have been linked to enhanced thermotolerance. In general, the kveik yeasts fell into statistical groupings between the WLP001/WLP002/WLP029 and WLP570 strains (Supplementary Data Sheet 2). We have observed heterozygous loss-of-function mutations in several genes relevant to thermotolerance, including *KEX1* (cell death protease; 4/6 sequenced kveik strains), *LRG1* (Rho1-specific GTPase-activating protein and negative regulator of PKC-controlled cell wall integrity pathway; 6/6 sequenced kveik strains), *SWP82* (member of the SWI/SNF chromatin remodeling complex; 1/6 sequenced kveik strains), *RPI1* (modulates cell wall integrity; 6/6 sequenced kveik strains), *IRA1/IRA2* (GTPase-activating proteins and inhibitory regulators of the RAS-cAMP pathway; 6/6 and 1/6 sequenced kveik strains, respectively), and *CDC25* (membrane bound guanine nucleotide exchange factor and activator of RAS-cAMP pathway; 4/6 sequenced kveik strains) (Jones et al., 1991; Lorberg et al., 2001; Puria et al., 2009; Wallace-Salinas et al., 2015; Satomura et al., 2016; Huang et al., 2018; Supplementary Table S5).

**Table 6:** Thermotolerance and ethanol tolerance in kveik yeasts.

	**Temperature** (**°C)**				**Ethanol (% v/v)**			
	**30**	**40**	**42**	**43**	**10**	**12**	**14**	**16**
WLP570	2.00	1.80	0.51	0.39	1.84	0.50	0.41	0.37
WLP001	1.93	0.14	0.13	0.12	0.80	0.48	0.34	0.14
WLP002	1.90	0.21	0.11	0.11	0.56	0.11	0.11	0.10
WLP029	1.96	0.17	0.11	0.11	0.50	0.40	0.10	0.10
Granvin 1	1.86	**1.53**	0.42	0.35	**1.18**	0.42	0.10	0.10
Granvin 2	1.92	**1.40**	**0.36**	**0.28**	**1.44**	0.55	0.45	0.25
Granvin 3	1.95	**1.53**	0.45	**0.31**	0.72	**0.27**	0.12	0.10
Granvin 4	1.87	**1.53**	0.23	**0.15**	0.70	0.38	**0.21**	0.10
Granvin 5	1.91	0.13	0.10	0.13	0.40	0.11	0.10	0.10
Granvin 6	1.84	1.74	0.41	0.40	**1.63**	0.46	0.42	0.19
Granvin 7	1.82	**0.70**	**0.31**	**0.25**	0.74	0.33	0.16	0.10
Granvin 8	1.84	0.14	0.13	0.13	0.62	0.21	0.10	0.10
Granvin 9	1.84	**0.84**	0.44	**0.22**	0.77	0.25	0.10	0.10
Hornindal 1	1.84	1.76	0.41	0.35	**1.39**	0.48	**0.30**	**0.27**
Hornindal 2	1.88	1.67	**0.33**	**0.26**	**1.12**	0.40	**0.32**	**0.22**
Hornindal 3	1.93	**1.49**	0.22	**0.19**	**1.47**	0.48	**0.27**	**0.29**
Joniskelis	1.88	**1.62**	0.56	**0.30**	1.70	0.62	**0.54**	0.37
Laerdal 1	1.83	**1.70**	0.48	0.44	1.79	0.50	0.40	0.33
Laerdal 2	1.86	**1.21**	0.45	0.33	**1.39**	0.47	0.39	**0.24**
Muri 1	1.96	**0.51**	**0.33**	0.30	0.93	0.47	0.49	**0.21**
Stordal Ebbegarden 1	1.81	**1.41**	**0.36**	**0.29**	0.73	0.47	0.47	0.34
Stordal Ebbegarden 2	1.91	**0.32**	**0.25**	**0.21**	0.72	0.39	0.27	0.10
Stordal Framgarden 1	1.97	1.64	**0.29**	**0.25**	**1.39**	0.60	0.41	0.32
Stordal Framgarden 2	1.84	1.72	**0.28**	**0.19**	**1.47**	0.61	0.44	0.33
Stranda	1.86	1.48	0.16	**0.18**	**1.14**	0.45	0.33	0.13
Sykkylven 1	1.87	1.78	0.46	**0.30**	1.70	0.51	**0.31**	**0.20**
Sykkylven 2	1.83	1.26	**0.26**	**0.26**	**1.01**	0.50	0.28	0.16
Voss 1	1.83	1.84	0.70	**0.30**	1.79	0.56	0.39	**0.22**
Voss 2	1.97	1.82	0.60	**0.24**	1.79	0.58	0.47	0.19

*High temperature and ethanol tolerance assays were performed as described in materials and methods. OD_600_ readings were obtained following 20 h of incubation in the specified conditions. Values represent the mean of biological replicates. Statistical analysis is available via Supplementary Data Sheet 2. Values marked in bold are significantly different from all controls (P < 0.05, one-way ANOVA with Tukey's post-hoc test)*.

We next investigated the ethanol tolerance of kveik yeasts in comparison to the control ale strains with ethanol tolerances available from the supplier (White Labs). Kveik and control strains were inoculated at 0.1 OD_600_ into media containing from 10 to 16% ethanol and grown aerobically for 20 h. Our control data were in line with the suppliers' broadly specified ethanol tolerances, e.g., WLP570 to “High–10 to 15%” and WLP002 to “Medium–5 to 10%” (Table 6). Interestingly WLP570, a Belgian-origin strain, showed high ethanol tolerance with evidence of growth up to 16% ethanol. Compared to the American, British and German-origin strains (WLP001, WLP002, WLP029, respectively) the kveik strains generally showed superior ethanol tolerance. 19/25 kveik strains at least doubled in density during the growth period at 14% ethanol, while 13/25 strains at least doubled in density during the growth period at 16% ethanol. Again, the kveik yeasts often fell into statistical groupings between the WLP001/WLP002/WLP029 and WLP570 strains (Supplementary Data Sheet 2). With exception to a number of strains originating from the Granvin sample, kveik yeasts display high levels of ethanol tolerance, suggesting that ethanol tolerance is generally conserved among kveik yeasts and may be a domestication signature of this yeast group. Supporting the phenotypic data, we observed a number of mutations relevant to ethanol tolerance in the sequenced kveik strains (Supplementary Table S5). Among these are *AGP2* (heterozygous, 6/6 strains), *PCA1* (heterozygous, 6/6 strains), and *VPS70* (heterozygous, 6/6 strains) (Teixeira et al., 2009; Voordeckers et al., 2015).

**Figure 5:**
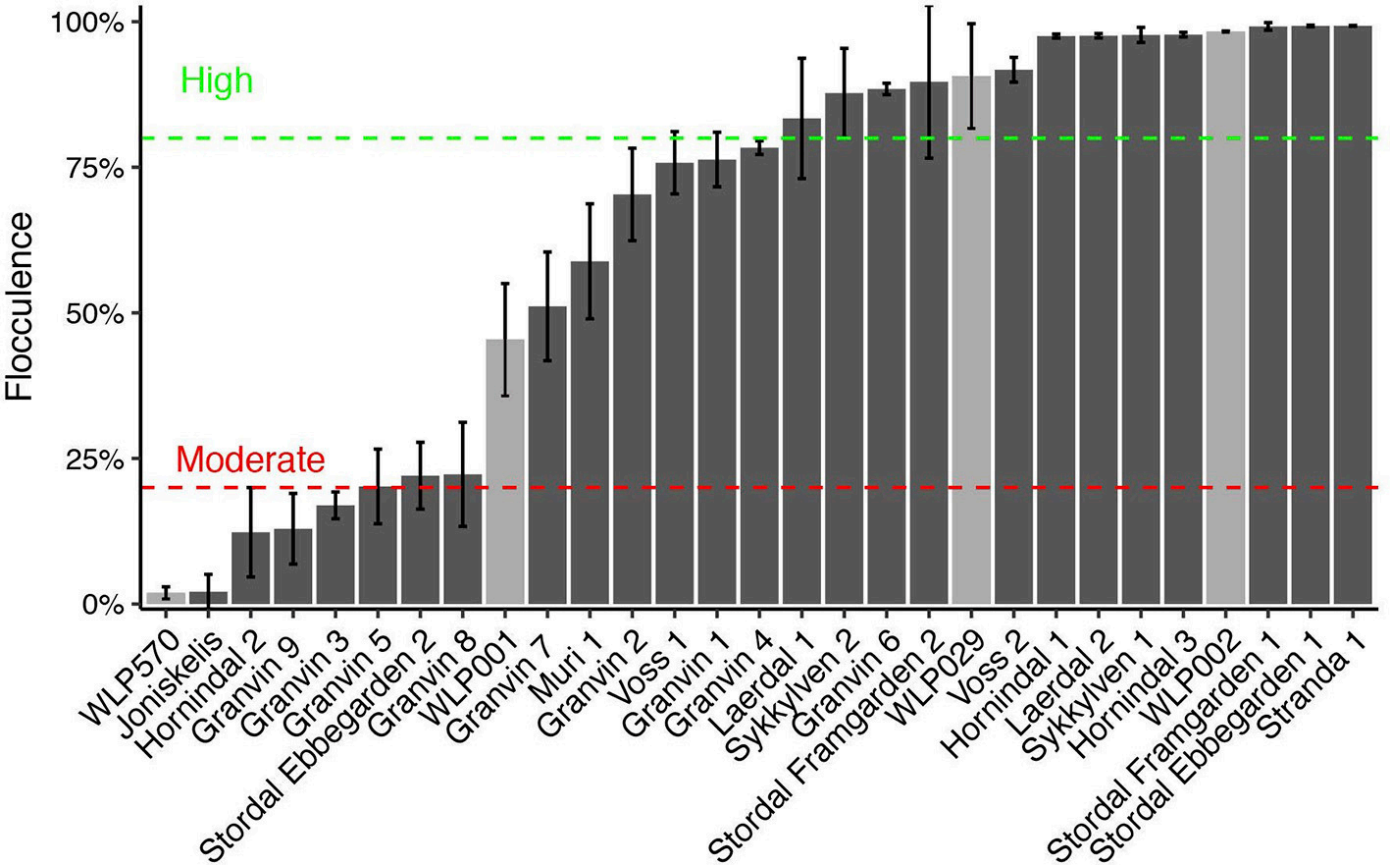
Flocculation capacity of kveik yeasts. Flocculation was assessed using the spectrophotometric absorbance methodology of ASBC Method Yeast-11. Values are expressed as %flocculance, with <20% representing non-flocculent yeasts, between 20 and 80% representing moderately flocculant yeast and >80% representing highly flocculant yeast. Strains are sorted in order of flocculance. Error bars represent SD, *n* = 3.

Flocculation is a hallmark of yeast domestication, as this property enhances the brewer's ability to harvest yeast via either top or bottom cropping in the fermenter. We assessed the flocculence of the kveik yeasts using the absorbance method of ASBC Yeast-11 Flocculence method of analysis (ASBC, 2011). The control strains produced expected flocculence values: for example, the Belgian strain (WLP570) is non-flocculant (2%) and the British strain (WLP002) is highly flocculant (98%) (Figure 5). We observed high levels of flocculation among the kveik yeasts, but this property was not universal: 12/24 strains had flocculence values >80% (highly flocculant), while others showed very low flocculance (<20%; 4 strains). Interestingly, in most kveik samples containing more than one strain, at least one of the strains showed high flocculation rates above 80% (Figure 5). It is possible that in the original kveik mixed *S. cerevisiae* cultures, the yeasts undergo co-flocculation and consequently some strains never developed or needed this function (Smukalla et al., 2008; Rossouw et al., 2015). Nonetheless, the high incidence of efficient flocculation among kveik yeasts is further support these yeasts have been domesticated. Copy number variations linked to flocculation genes (*FLO*) are common among domesticated yeasts (Dunn et al., 2012; Bergström et al., 2014; Gallone et al., 2016; Steenwyk and Rokas, 2017). Upon examination of the WGS data, we observed a high degree of copy number variation in *FLO* genes in the sequenced kveik strains (Table 7). Notably, the only strain with very low flocculence analyzed with whole genome sequencing (“Hornindal 2”; 12.3%) had a complete deletion of *FLO1*, known to be a critical gene conferring the flocculent phenotype (Vidgren and Londesborough, 2011). The flocculence of this strain was significantly lower (*P* < 0.05) when compared to the Hornindal 1 strain. It is also worth noting that all kveik yeasts sequenced carry a 425A>G SNP in *FLO8* which causes a lost stop codon, restoring the functionality of *FLO8*, which is inactive in the S288c reference strain (Liu et al., 1996).

**Table 7:** Estimated copy number variation among flocculation (*FLO*) genes in kveik.

**Strain**	***FLO1***	***FLO5***	***FLO8***	***FLO9***	***FLO10***	***FLO11***
Voss 1	1	0	4	2	0	5
Laerdal 2	3	1	4	2	0	2
Hornindal 1	2	1	4	2	0	5
Stordal Ebbegarden 1	1	1	4	2	0	1
Granvin 1	1	0	4	2	0	5
Hornindal 2	0	1	4	2	0	1

## Discussion

Here we present evidence which suggests kveik yeasts obtained from Norwegian farmhouse brewers represent a previously undiscovered group of genetically distinct and domesticated beer yeasts, and that these yeasts have promising beer production attributes (Almeida et al., 2015; Baker et al., 2015; Gallone et al., 2016; Gonçalves et al., 2016). Our PCR fingerprint data suggested kveik yeast strains form a genetically distinct group of ale yeasts. Moreover, whole genome sequencing analysis of a representative group of 6 strains shows that kveik yeasts form a distinct group likely related to the “Beer 1” clade but with possible mixed ancestry when the separate haplotypes of the kveik yeasts are analyzed separately. The apparent conserved mixed ancestry of kveik is interesting given that mosaic/mixed-origin beer yeasts are not particularly common among either major beer yeast group (Gallone et al., 2016). Importantly, our analysis of Norwegian kveik yeasts suggests that the high-frequency production pressure of industrialization may not be necessary for domestication of brewing yeasts.

Our investigation of the beer production attributes with small-scale fermentation trials, phenotypic screens and genome sequencing revealed the majority of the Norwegian kveik yeasts metabolize wort sugars quickly (with related CNVs in maltose-relevant genes), are POF- (with loss-of-function mutations in *PAD1* and *FDC1*), flocculate efficiently (with CNVs in the *FLO* and related genes), and are highly ethanol tolerant and thermotolerant (typically polygenic traits). The domestication phenotypes and genomic domestication markers in kveik largely line up with those of previously analyzed domesticated beer yeasts (Gallone et al., 2016; Gonçalves et al., 2016). Thus, it appears that kveik have been domesticated in a similar manner to modern industrialized ale yeasts. The increased production rates of early industrial breweries in the seventeenth to eighteenth century was previously proposed to provide the foundation for beer yeast domestication (Gallone et al., 2016). Here we show kveik yeasts, surprisingly, have similar adaptation characteristics to the beer fermentation environment despite presumably being domesticated by farmhouse brewers without the high-frequency production pressure of an industrial brewing environment (Gallone et al., 2016). Thus, it is possible that the high frequency beer production associated with industrialization was not the only mechanism of adaptation resulting in the domesticated beer yeasts used today. Whether or not similar, small scale brewing practices analogous to the Norwegian farmhouse brewing culture, resulted in the domestication of yeast strains in Beer 1 predating industrialization, is currently unknown. As more yeast genomic data become available, it may be possible to identify yeasts which are more closely related to kveik and better understand the timeline of domestication for these yeasts and for other domesticated beer yeasts.

Approximately one third of the kveik yeasts did not flocculate with high efficiency. This may be influenced by the procedure used by farmhouse brewers to harvest yeast for repitching, including harvesting at least some of the top-fermenting yeast cells where the evolutionary pressure to flocculate would be less. It is therefore not surprising that some kveik strains flocculate less efficiently than others. However, kveik may present a new model for understanding yeast co-flocculation given the ability for high flocculation in some but not all members of a mixed yeast culture (e.g., the Hornindal culture) (Nishihara et al., 2000; Stewart, 2015).

Wort fermentations revealed that kveik strains produce a range of fruity esters, with ethyl caproate, ethyl caprylate, ethyl decanoate, and phenethyl acetate present above detection threshold (Table 3), indicating that these yeasts can be used to produce beers with fruity character. How kveik yeasts compare to a broader range of industrial beer yeasts in terms of diversity and intensity of flavor production is currently unknown and is a limitation of the present study. We have shown the kveik ale yeasts have a broad range of wort attenuation values. As these yeasts are POF-, a desirable trait for the majority of beer styles (McMurrough et al., 1996), they also could have broad utility for ale production, with selection by the brewer in accordance with desired attenuation target values and flavor profiles.

Strikingly, our phenotypic screening revealed the favorable thermotolerance and ethanol tolerance of these yeasts in comparison to known domesticated beer yeasts. Long-term heat adaptation is particularly relevant to fermentation processes performed at elevated temperatures, including those used for industrial bioethanol production. Multiple molecular and cellular processes and targets have been identified in the adaptation of yeast to heat. A prior study investigating the adaptation of yeast to ~40°C over a prolonged period of time, identified SNPs in genes related to DNA repair, replication, membrane composition and membrane structure as specific genetic markers of thermotolerance (Caspeta et al., 2013). Similarly, we identify SNPs in: *CDC25, IRA1*, and *IRA2*, which are genes that regulate the RAS/cAMP/PKA pathway; *RPI1* and *LRG1*, which impacts cell wall integrity; and *KEX1* and *SWP82* (Puria et al., 2009; Wallace-Salinas et al., 2015; Peeters et al., 2017; Huang et al., 2018). These mutations could aid thermotolerance in kveik and be future routes for development of thermotolerant yeasts. This characteristic also has potential application in brewing, as wort inoculation at higher temperatures (>30°C) without compromise in flavor could help limit the expensive cooling needed to manage wort fermentation temperatures that are typically controlled at 18–22°C for ale fermentations (Hill, 2015).

We also demonstrate that ethanol tolerance, known to be a polygenic and genetically complex trait involving multiple alleles, is a common adaptation of kveik yeasts. While single genetic alterations can incrementally increase ethanol tolerance, it does not approach that of the polygenic/multiallelic phenotype (Lam et al., 2014; Snoek et al., 2016). High ethanol environments generally disrupt cell membrane structure and function, and impact protein folding. Not surprisingly, genes linked to ethanol tolerance are often associated with: stabilizing cell walls and cell membranes; increasing the protein folding capacity; maintaining the electrochemical gradient across the plasma membrane; and maintaining vacuolar function to mention a few (Lam et al., 2014; Snoek et al., 2016). Remarkably, almost one third of the kveik yeasts reported here could grow in the presence of 16% ethanol. Correspondingly, we observed mutations in genes linked to ethanol tolerance among the sequenced kveik strains, comprising *AGP2, PCA1*, and *VPS70* (Teixeira et al., 2009; Voordeckers et al., 2015). Interestingly, these mutations were always heterozygous. Given the ethanol and high temperature tolerances of kveik yeasts, it is possible these yeasts could benefit the distillation and bioethanol industries where these traits are desired (Caspeta et al., 2016).

It is now known that a broader selection of traditional Norwegian kveik yeasts are still in existence, and it is possible that other domesticated or “landrace yeasts” may exist in other geographic locations beyond Norway. Whole genome sequencing of additional kveik yeasts could better support geographical subgroups suggested in the present study. Furthermore, further detailed analysis into the individual kveik cultures (for example, screening more colonies) may reveal greater strain diversity than evident here. The apparent mosaic nature of the kveik genomes also warrants further investigation. To elucidate the ancestry of the kveik strains in more detail, one could apply the use of long read sequencing to improve the quality and length of the haplotype blocks during phasing and expanding the genome data set, e.g., with the recently published 1,011 yeast genomes (Peter et al., 2018), used for phylogenetic and population structure analysis. It is possible that through more detailed phenotypic screening and sequencing, particularly using long-read technology, a wider range of such yeasts may result in an expanded understanding of beer yeast domestication given the noted differences between farmhouse ale production (infrequent, non-commercial) vs. industrial ale production (frequent, commercial).

## Data availability statement

The whole genome sequence datasets generated for this study can be found in the NCBI BioProject number PRJNA473622 (https://www.ncbi.nlm.nih.gov/bioproject/PRJNA473622).

## Author contributions

CT, RP, and KK conducted the experiments described in this study. KK performed bioinformatic analysis of the whole genome sequence data. RP, CT, and GM designed the experiments. LG contributed to introductory materials and supplied yeast cultures. RP, KK, and GM wrote the manuscript. All authors read and approved the final manuscript.

### Conflict of interest statement

The authors declare that the research was conducted in the absence of any commercial or financial relationships that could be construed as a potential conflict of interest.
