# Supplementary material for: Traditional Norwegian Kveik Are a Genetically Distinct Group of Domesticated Saccharomyces cerevisiae Brewing Yeasts
Source: Front Microbiol. 2018 Sep 12;9:2137. doi: 10.3389/fmicb.2018.02137 (PMC6145013; doi:10.3389/fmicb.2018.02137)
Supplement: Supplementary file 1 [file Table_1.DOCX]

**Supplementary Table S1.** Kveik yeast cultures, corresponding NCYC catalogue numbers, and reported beer inoculation temperature.

| Kveik yeast culture | NCYC catalogue number | Inoculation temperature | Source |
| --- | --- | --- | --- |
| Stordal Ebbegarden | 4224^1^ | 28 °C | Phone interview with Jens Aage Øvrebust, October 2016 |
| Stordal Framgarden | 4170^1^ | 30 °C | Phone interview with Jens Aage Øvrebust, October 2016 |
| Granvin | Not assigned ^2^ | 30 °C | Phone interview with Tor Ølver Helland, 2017-10-27 |
| Hornindal | 4051^1^ | 30 °C | Brewing session with Terje Raftevold in Innvik, 2015-07-04 |
| Joniškėlis | 4207^1^ | 35 °C | Interview with Julius Simonaitis, conducted by Simonas Gutautas in Joniškėlis, 2016-03-24 |
| Lærdal | Not assigned ^2^ | 30 °C | Phone interview with Dagfinn Wendelbo, 2017-10-30 |
| Muri | 4045^1^ | 32 °C | Phone interview with Bjarne Muri, 2016-12-30 |
| Stranda | 4021^1^ | 30 °C | Facebook message from Stein Langlo, 2018-03-04 |
| Sykkylven | 4171^1^ | 30 °C | Facebook message from Sigurd Johan Saure, January 2016 |
| Voss | 3995^1^ | 39 °C | Brewing session with Sigmund Gjernes in Voss, 2014-05-24 |

^1^  Original mixed yeast culture.
^2^ Sample submitted but not yet assigned a catalogue number.
