# Supplementary material for: Traditional Norwegian Kveik Are a Genetically Distinct Group of Domesticated Saccharomyces cerevisiae Brewing Yeasts
Source: Front Microbiol. 2018 Sep 12;9:2137. doi: 10.3389/fmicb.2018.02137 (PMC6145013; doi:10.3389/fmicb.2018.02137)
Supplement: Supplementary file 2 [file Table_2.DOCX]

**Supplementary Table S2.** Summary of the software and parameters used for whole genome sequence analysis. Software were run with default parameters if none are given under the ‘Parameters’ column.

| **Software** | **Version** | **Parameters** |
| --- | --- | --- |
| BCFtools | 1.2 |  |
| BEDTools | 2.26.0 |  |
| Control-FREEC | 11.0 | ploidy = 4  breakPointThreshold = .6  window = 250  telocentromeric=7000 |
| FastQC | 0.11.5 |  |
| fastStructure | 1.0 | -K 11 |
| FreeBayes | 1.1.0-46-g8d2b3a0 | -p 4 |
| IQ-TREE | 1.5.5 | -bb 1000  –m GTR+R4 |
| NASP | 1.0.0 |  |
| Qualimap | 2.2.1 |  |
| Plink | 1.9 | --indep-pairwise 50 5 0.5  --maf 0.05 |
| SAMtools | 1.2 | -bq 50 |
| SnpEff | 1.2 | -no-downstream  -no-upstream  -no-intergenic |
| SpeedSeq | 0.1.0 |  |
| STRUCTURE | 2.3.4 | -K 11  BURNIN 100000  NUMREPS 100000 |
| structure_threader | 1.2.4 | -K 11  -R 10 |
| Trimmomatic | 0.36 | -phred33  ILLUMINACLIP:[adapter.fa]:2:30:10  SLIDINGWINDOW:5:20  MINLEN:36 |
| VCFtools | 0.1.15 | --maf 0.01 |
| WhatsHap | 0.14.1 |  |
