# Supplementary material for: Traditional Norwegian Kveik Are a Genetically Distinct Group of Domesticated Saccharomyces cerevisiae Brewing Yeasts
Source: Front Microbiol. 2018 Sep 12;9:2137. doi: 10.3389/fmicb.2018.02137 (PMC6145013; doi:10.3389/fmicb.2018.02137)
Supplement: Supplementary file 3 [file Table_3.DOCX]

**Supplementary Table S3**. Distance between populations. Nucleotide substitutions (10^-3^) per site across the genome.

|  | **Beer 1 - US** | **Beer 1 - UK** | **Beer 1 – Bel/Ger** | **Beer 2** | **Kveik** | **Mixed** | **Mosaic** | **Wine** |
| --- | --- | --- | --- | --- | --- | --- | --- | --- |
| **Asia** | 2.71 | 2.64 | 2.60 | 2.90 | **2.77** | 2.28 | 2.66 | 3.16 |
| **Beer 1 - US** |  | 1.36 | 1.56 | 1.91 | **2.55** | 1.79 | 1.90 | 1.75 |
| **Beer 1 - UK** |  |  | 1.48 | 1.85 | **2.49** | 1.68 | 1.82 | 1.69 |
| **Beer 1 – Bel/Ger** |  |  |  | 1.89 | **2.50** | 1.71 | 1.89 | 1.76 |
| **Beer2** |  |  |  |  | **2.95** | 1.81 | 1.83 | 1.23 |
| **Kveik** |  |  |  |  |  | **2.50** | **2.87** | **2.98** |
| **Mixed** |  |  |  |  |  |  | 1.85 | 1.70 |
| **Mosaic** |  |  |  |  |  |  |  | 1.71 |
