# Supplementary material for: Traditional Norwegian Kveik Are a Genetically Distinct Group of Domesticated Saccharomyces cerevisiae Brewing Yeasts
Source: Front Microbiol. 2018 Sep 12;9:2137. doi: 10.3389/fmicb.2018.02137 (PMC6145013; doi:10.3389/fmicb.2018.02137)
Supplement: Supplementary file 4 [file Table_4.DOCX]

**Supplementary Table S4**. Distance within populations. Nucleotide substitutions (10^-3^) per site across the genome.

| **Asia** | **Beer 1 – US** | **Beer 1 - UK** | **Beer 1 – Bel/Ger** | **Beer 2** | **Kveik** | **Mixed** | **Mosaic** | **Wine** |
| --- | --- | --- | --- | --- | --- | --- | --- | --- |
| 0.68 | 0.34 | 0.74 | 0.88 | 1.01 | **1.65** | 1.03 | 2.00 | 0.63 |
