# Supplementary material for: Traditional Norwegian Kveik Are a Genetically Distinct Group of Domesticated Saccharomyces cerevisiae Brewing Yeasts
Source: Front Microbiol. 2018 Sep 12;9:2137. doi: 10.3389/fmicb.2018.02137 (PMC6145013; doi:10.3389/fmicb.2018.02137)
Supplement: Supplementary file 5 [file Table_5.DOCX]

**Supplementary Table S5.** A selection of other non-synonymous SNPs unique to the six ‘kveik’ strains (i.e. they were not detected in any of the 157 genome assemblies in BioProject PRJNA323691 (Gallone et al., 2016)).

| **Gene** | **Description** | **Nucleotide (and amino acid) change** | **Granvin 1** | **Hornindal 1** | **Hornindal 2** | **Laerdal 2** | **Stordal Ebbegarden 1** | **Voss 1** |
| --- | --- | --- | --- | --- | --- | --- | --- | --- |
| *AGP2 (YBR132C)* | Plasma membrane regulator; shown to be associated with ethanol tolerance. (Teixeira et al., 2009) | 772C>T  (Arg258Cys) | **0/0/1/1** | **0/0/0/1** | **0/0/0/1** | **0/0/1/1** | **0/0/0/1** | **0/0/1/1** |
| *ATF1 (YOR377W)* | Alcohol acetyltransferase; responsible for volatile acetate ester production during fermentation. | 1577A>G  (526 stop lost) | 0/0/0/0 | 0/0/0/0 | 0/0/0/0 | **1/1/1/1** | 0/0/0/0 | **0/0/1/1** |
| *ATF2 (YGR177C)* | Alcohol acetyltransferase; responsible for volatile acetate ester production during fermentation. | 1275delG  (Val427fs) | 0/0/0/0 | **0/0/0/1** | 0/0/0/0 | 0/0/0/0 | **0/1/1/1** | 0/0/0/0 |
| *CDC25 (YLR310C)* | Membrane bound guanine nucleotide exchange factor; mutations in *CDC25* have been linked to increased heat tolerance. (Satomura et al., 2016) | 1360T>C  (Ser454Pro) | 0/0/0/0 | **0/0/1/1** | 0/0/0/0 | 0/0/0/0 | 0/0/0/0 | 0/0/0/0 |
|  |  | 3830C>T  (Pro1277Leu) | 0/0/0/0 | 0/0/0/0 | **0/0/1/1** | 0/0/0/0 | **0/0/0/1** | 0/0/0/0 |
|  |  | 3995G>A  (Arg1332Lys) | **0/1/1/1** | **0/0/1/1** | 0/0/0/0 | 0/0/0/0 | 0/0/0/0 | 0/0/0/0 |
| *IRA1 (YBR140C)* | GTPase-activating protein; Inhibitory regulator of the RAS-cAMP pathway. | 6604G>A  (Glu2202Lys) | **0/0/0/1** | **0/0/0/1** | **0/0/1/1** | **0/0/1/1** | **0/0/0/1** | **0/0/1/1** |
| *IRA2 (YOL081W)* | GTPase-activating protein; Inhibitory regulator of the RAS-cAMP pathway. | 864T>G  (Tyr288*) | 0/0/0/0 | 0/0/0/0 | 0/0/0/0 | 0/0/0/0 | **0/0/0/1** | 0/0/0/0 |
| *KEX1 (YGL203C)* | Cell death protease; Deletion enhances heat tolerance. (Huang et al., 2018) | 340T>C (Phe114Leu) | **0/0/0/1** | **0/0/0/1** | 0/0/0/0 | **0/0/1/1** | 0/0/0/0 | **0/0/0/1** |
|  |  | 923C>T (Ser308Leu) | **0/0/1/1** | **0/0/0/1** | 0/0/0/0 | **0/0/0/1** | 0/0/0/0 | 0/0/0/0 |
|  |  | 1336G>A (Asp446Asn) | 0/0/0/0 | 0/0/0/0 | 0/0/0/0 | 0/0/0/0 | 0/0/0/0 | **0/0/0/1** |
| *LRG1 (YDL240W)* | GTPase-activating protein; Deletion enhances heat tolerance. (Huang et al., 2018) | 257G>A (Cys86Tyr) | 0/0/0/0 | 0/0/0/0 | 0/0/0/0 | 0/0/0/0 | 0/0/0/0 | **0/0/0/1** |
|  |  | 974T>C (Leu325Pro) | **0/0/0/1** | **0/0/0/1** | 0/0/0/0 | **0/0/0/1** | 0/0/0/0 | 0/0/0/0 |
|  |  | 1565C>T (Ser522Phe) | **0/0/0/1** | **0/0/0/1** | 0/0/0/0 | 0/0/0/0 | 0/0/0/0 | **0/0/0/1** |
|  |  | 2519T>C (Ile840Thr) | 0/0/0/0 | 0/0/0/0 | **0/1/1/1** | 0/0/0/0 | 0/0/0/0 | 0/0/0/0 |
| *PCA1 (YBR295W)* | Cadmium transporting ATPase; mutations in *PCA1* linked to increased ethanol tolerance. (Voordeckers et al., 2015) | 1022-1045 deletion (Lys341fs) | **0/0/1/1** | **0/0/1/1** | **0/0/1/1** | **0/0/1/1** | **0/0/1/1** | **0/0/0/1** |
| *RMR1 (YGL250W)* | Protein required for meiotic recombination and gene conversion. | 726A>T  (242 stop lost) | **0/0/1/1** | **0/1/1/1** | **0/0/0/1** | **0/0/1/1** | **1/1/1/1** | **0/1/1/1** |
| *RPI1 (YIL119C)* | Transcription factor; mediates fermentation stress tolerance by modulating cell wall integrity. | 820A>G  (Thr274Ala) | **0/0/1/1** | **0/0/1/1** | **0/0/0/1** | **0/0/1/1** | **0/0/1/1** | **0/0/0/1** |
| *SAS2 (YMR127C)* | Acetyltransferase; Deletion enhances flocculation and maltose fermentation. (Rodriguez et al., 2014) | 986T>G  (Leu329*) | 0/0/0/0 | 0/0/0/0 | **0/0/1/1** | 0/0/0/0 | **0/0/0/1** | 0/0/0/0 |
| *SWP82*  *(YFL049W)* | Member of the SWI/SNF chromatin remodeling complex; Deletion enhances heat tolerance. (Huang et al., 2018) | 236C>T (Thr79Met) | 0/0/0/0 | 0/0/0/0 | **0/0/0/1** | 0/0/0/0 | 0/0/0/0 | 0/0/0/0 |
|  |  | 436G>T (Glu146*) | 0/0/0/0 | 0/0/0/0 | **0/0/0/1** | 0/0/0/0 | 0/0/0/0 | 0/0/0/0 |
|  |  | 1076G>A (Gly359Asp) | 0/0/0/0 | 0/0/0/0 | **0/0/0/1** | 0/0/0/0 | 0/0/0/0 | 0/0/0/0 |
| *TGL5 (YOR081C)* | Triacylglycerol lipase; releases fatty acids from neutral lipid triacylglycerols. | 2241complex  (Gln748fs) | **0/0/0/1** | **0/0/0/1** | **0/1/1/1** | **0/0/0/1** | **0/1/1/1** | **0/0/0/1** |
| *VPS70 (YJR126C)* | Involved in vacuolar protein sorting; mutations in *VPS70* linked to increased ethanol tolerance. (Voordeckers et al., 2015) | 1522G>A  (Ala508Thr) | **0/0/0/1** | **0/0/0/1** | **0/0/1/1** | **0/1/1/1** | **0/0/0/1** | **0/0/1/1** |
